# Group based video-conferencing for adults with depression: findings from a user-led qualitative data analysis using participatory theme elicitation

**DOI:** 10.1186/s40900-019-0173-z

**Published:** 2019-12-05

**Authors:** Paul Best, Tracey McConnell, Gavin Davidson, Jennifer Badham, Ruth D. Neill

**Affiliations:** 1 School of Social Sciences Education and Social Work. 6 College Park, Queen’s University Belfast, Belfast, UK; 2 Centre for Public Health, School of Medicine, Dentistry and Biomedical Sciences, Royal Victoria Hospital, Queen’s University Belfast, Belfast, UK; 3 Centre for Evidence and Social Innovation, Queen’s University Belfast, Belfast, UK

**Keywords:** Participatory theme elicitation, User involvement, Participatory analysis

## Abstract

**Background:**

Accessing support services for depression has been historically difficult given the societal stigma that exists regarding the condition. Recent advances in digital technologies continue to be postulated as a potential panacea yet the results from research trials have been mixed with a range of effect sizes.

**Methods:**

This article offers a different perspective by presenting a panel of end users (co-researchers) with qualitative interview data (*n* = 8) taken from a feasibility RCT of a group based video-conferencing service for depressed adults. The co-researcher panel were introduced to a new method of participatory data analysis known as Participatory Theme Elicitation (PTE). This method involves using network analysis techniques to create groupings and visual diagrams in order to support the generation of themes and minimise scientific researcher input/influence.

**Results:**

Co-researchers reported that while VC based interventions appeared convenient, accessible and relatively low cost - additional training and support should be offered to improve uptake and retention. In addition, co-researchers suggested that further exploration is needed regarding the level of self-awareness one feels in a group based VC environment and whether this facilitates disclosure (through disinhibition) or increases anxiety.

**Conclusion:**

The findings presented here appear to support existing (researcher and academic-led) literature in the field as well as suggest new areas for investigation. By presenting data generated solely by co-researchers, this article also adds to the evidence surrounding participatory analysis methods - particularly the growing need for robust approaches that are accessible and less time-consuming than those currently available.

**Trial registration number:**

NCT03288506 (Clinicaltrials.gov) 20th Sept 2017 https://clinicaltrials.gov/ct2/show/NCT03288506

## Plain English summary

Video-conferencing technology, such as Skype may be a useful substitute for those who find it difficult to attend face-to-face services for mental health issues. The interview data presented here is gathered from individuals who took part in an 8-week online support group intervention for depression using video-conferencing (VC) technology. A unique aspect of this study is that the interview data was analysed by members of the public (co-researchers) who had previously accessed face-to-face support for depression. This was achieved using a new method of participatory analysis known as Participatory Theme Elicitation (PTE). The co-researchers generated themes that were both consistent with previous literature as well as revealing new insights by drawing on their unique experiences and perspectives. Key findings suggest that VC technology is accessible and relatively low cost with some data showing how the service improved mental health. Nonetheless, co-researchers suggested attention should be paid to the increased sense of self-awareness one may feel within an online group setting and whether this discourages participation and increases anxiety.

## Introduction

The World Health Organisation (WHO) has reported that ‘depression is now the leading cause of ill health and disability worldwide’ [1]. Depression is a common mental disorder affecting approximately 300 million people globally [1]. Since 2015, there has been a marked increase in the prevalence of depression with some data showing a global uplift of 18% [1]. Within the UK, it is estimated that nearly a fifth (19%) of all adults’ experience depression or anxiety [2]. Common symptoms of depression include a loss of energy; a change in appetite; sleeping more or less; anxiety; reduced concentration; indecisiveness; restlessness; feelings of worthlessness, guilt, or hopelessness; and thoughts of self-harm or suicide [3]. Accessing support services for depression has been historically difficult given the societal stigma that exists regarding the condition [4, 5]. However, recent technological advances are facilitating a shift towards more online forms of support for mental health issues – particularly given the anonymity and confidentiality benefits [6].

The Priory Group [7] estimates that in the UK alone ‘depression’ is Googled every 2 s with over 20 searches per minute for anxiety and stress. This online approach to accessing information appears to have empowered individuals to take more control over the health advice they receive and it has been postulated to be beneficial for people who are reluctant to seek help elsewhere. As such, online services are beginning to gain more attention within both the academic and health services world [8].

One of the more promising forms of online support is through the use of Video Conferencing VC technology [9–11] with a recent Cochrane review showing how VC based therapy can be effective for mental health issues [12]. Given the ubiquitous nature of online technologies, VC services are easily available and accessible, which may enable preventative processes to occur sooner. Moreover, there is also evidence to support reduced costs from using VC services e.g. in relation to home care and access to on-call hospital specialists [13]. Several studies have shown the mental health benefits of VC as comparable with face-to-face therapy [14–16]. However, the acceptability of this approach may depend on factors, such as age and technical competence [17]. After reviewing the outcomes of VC based treatment for clients with bulimia, Simpson et al. [18] (p.237) asserted “the distance and space provided by video therapy may have helped these clients to engage in treatment to a greater extent than would have been possible face-to face”. However, in spite of this evidence base, VC services for mental health are not widely available. As a result, the full risks and benefits for clinical purposes are unknown [19]. To date, the literature has shown some evidence of the potential for VC to be an effective treatment of mental health conditions as well as increasing engagement for groups who would otherwise struggle to do so using face-to-face services. Given the ubiquitous nature of online technologies, VC services are easily available and accessible which may enable preventative processes to occur sooner.

This article presents user-led qualitative data analyses from the Developing E-health Services (DES study) - an online group-based intervention for depression using video-conferencing (VC) technology. A unique aspect of this work is that the analysis presented here has been generated entirely by end users through an innovative network approach called Participatory Theme Elicitation (PTE), which is designed to make user involvement in data analysis more accessible for both users and researchers. The network analysis combines individually generated sets of themes into a combined set of themes and presents the results visually to support further discussion.

### Participatory data analyses

Stevenson and Taylor [21] highlight various gaps and barriers regarding meaningful involvement of users in the research process. Participatory data analysis, in particular, is one such area which requires further attention, having been largely neglected or under-reported. Participatory data analysis can be defined simply as involving users and key stakeholders in the data analysis phase of the research [22]. The benefits of this approach may include the potential for unique insider knowledge and more tailored solutions to complex problems [23–26]. One study of marginalised women in Canada developed a five step approach to group based participatory analysis [27] and noted that successful participatory approaches should be group oriented, engaging and facilitate user understanding. More recently, a study by Dunn [28] provided young people the opportunity to be involved in data synthesis as project co-researchers. As part of this work, they completed drawings, posters, maps and time-lines and attended a one-day workshop to generate recommendations for the programme. However, Dunn [28] cautiously noted that this approach enabled a more ‘careful synthesis’ of study findings rather than a complex analysis of the data. Others have gone further, claiming that involving children and young people in data analysis one uncovers a fresher perspective, possibly thinking outside of the normal research constraints and providing a more insightful and realistic viewpoint [29–31].

While there is evidence to suggest that participatory data analysis can be beneficial, the complexities and practicalities involved have meant that it is often under-utilised within research. Participatory approaches are often viewed as time-consuming [27, 30–32] and incorporating users in data analysis requires training and additional resources. The inexperience of co-researchers can also affect the reliability and validity of the study outcomes and themes identified [21, 30]. Best et al. [24] suggest that participatory approaches have struggled to establish methodological rigor and are therefore less likely to be adopted by others. Others have called for clearer direction and guidelines to be established [21, 31]. As such, robust, user led analysis of research data is sparse. In an effort to address this, the current study utilises PTE, an accessible and previously tested approach for the user-led analysis of qualitative data which involves a partnership with those who have lived experience of using services for depression. PTE draws upon previous work in a number of related areas [33–37] and the network techniques used within PTE serve to limit the potential for researcher bias and provide a useful starting point for co-researchers to begin analysing and interpreting qualitative data.

### Aims and objectives

#### Aim

To present the user-led analysis of qualitative process evaluation data from an online (group-based) intervention for depression using video-conferencing technology.

### Objectives

- To explore participants’ and facilitators’ experiences of using and delivering an online group based support service for depression
- To identify the key benefits, challenges and areas for improvement for the online service

## Methods

### Context

The DES Study (ClinicalTrials.gov Trial Number: NCT03288506).

The DES project was informed by the MRC framework for intervention development [38] and used a feasibility study design. The intervention was delivered over an 8-week period by a local mental health charity who developed an online version of their current face-to-face support group. Online groups took place on a weekly basis and lasted one hour. There was no control group. The qualitative data used in the current study was gathered at the end of the intervention (Week 8). Interviews were conducted either face-to-face or online (via Skype) and lasted between 20mins – 45mins. Audio recordings were taken and these were transcribed verbatim and anonymised. Ethical approval for the current study was approved by the University’s Ethics Committee.

### Participant recruitment (DES study)

Participants for the DES study were recruited via online registration and the service was promoted through local radio, newspaper and social media. Inclusion criteria were that participants are 18 years or older, not currently a user of the service provider’s face-to-face group(s) and not actively suicidal. Consent was obtained via an online form. Two online groups completed the 8-week intervention with semi-structured interviews conducted with five participants and three group facilitators across both groups (*n* = 8). Further details on recruitment, retention and outcomes for the DES study are published elsewhere [20].

### Co-researcher recruitment

The PTE method involves five key steps with co-researchers required for Steps Two, Three and Five (see Table 1). A convenience sample approach [39] was used to recruit co-researchers to undertake the PTE method. This was achieved through contacts at two local mental health charities. Information packs and consent forms were provided to individuals that showed an interest in taking part. Inclusion criteria for co-researchers was having previous experience of using services for depression and no prior knowledge of the online service. The research team liaised closely with staff in both those charity organisations to ensure that potential co-researchers were not actively suicidal and their mental health had been relatively stable for a period of six months or more. Nine co-researchers signed up to take part (*n* = 9). Ages ranged from 20 to 63, with three males and six females.

**Table 1:** Overview and the PTE five step process

PTE five step process*				
Step 1	Step 2	Step 3	Step 4	Step 5
*Data Selection*	*Capacity Building*	*Data Sorting*	*Data Grouping*	*Analysis and Interpretation*
Selection of representative quotes from qualitative data	Training of co-researchers	Sorting quotes into piles	Network analysis of user piles	Group discussion and review of network diagram and groupings
Time taken to complete: 2-3 h**	*Time taken to complete: 2 h*	*Time taken to complete: 2 h*	*Time taken to complete: 5–10 min*	*Time taken to complete: 2 h*

*There are no prescribed time limits within any of the five steps

**These times are representative of the current project only but may serve as a useful guide

#### Participatory theme elicitation

The general approach of PTE is that co-researchers independently sort qualitative data items into groups based on their similarity. These separate perspectives on similarity are combined into an overall set of groups. The overall groups are used to stimulate discussion about themes in the dataset (see [20] for a more detailed description of the methodology).

**Fig. 1:**
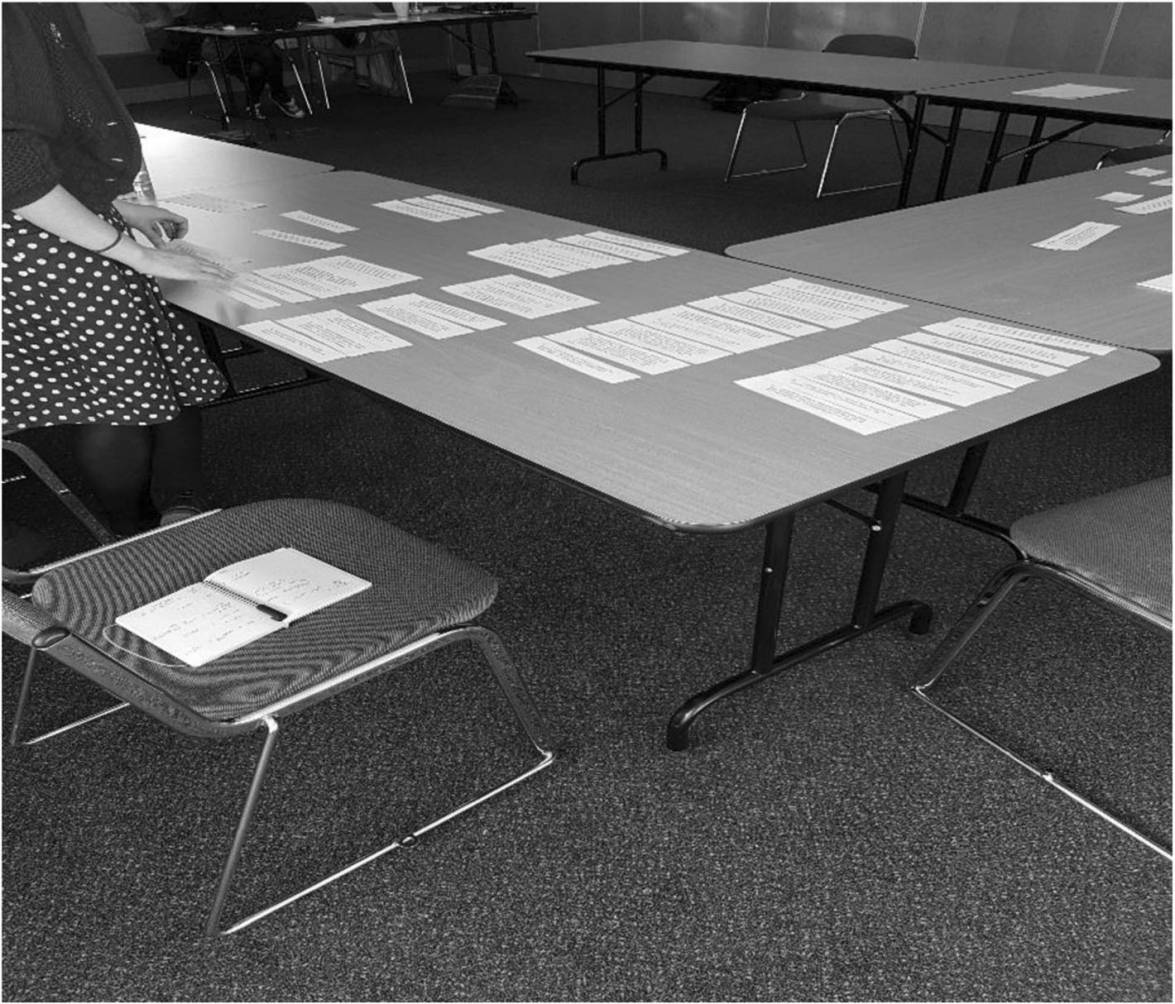
Photo of Co-researchers undertaking the sorting task

The first step within the PTE method is data selection. This involved a member of the research team (TC) and a member of the DES study’s user advisory group (CC) selecting standalone quotes from the qualitative transcripts and cutting them into strips (Fig. 1). So as to minimise undue influence, no guidance was given on quote selection beyond its ability to be read without context and that the final list represented the full range of discussion within the qualitative data (in so far as was practical). This produced a list of 49 quotes.

The second step was capacity building. Co-researchers attended two, 60-min training sessions covering (1) an overview of the DES Project; (2) an overview of qualitative and quantitative research; (3) an overview of PTE and user involvement within research and (4) sorting techniques (Step 3) used to group data together. Training took place back-to-back although not all co-researchers attended the same session (three were delivered in total). For the most part the training was interactive and included video content, images and a short quiz. Co-researchers were then given an information pack that included the 49 quotes, a blank sheet of paper, pens and instructions regarding the sorting task (Step 3).

**Fig. 2:**
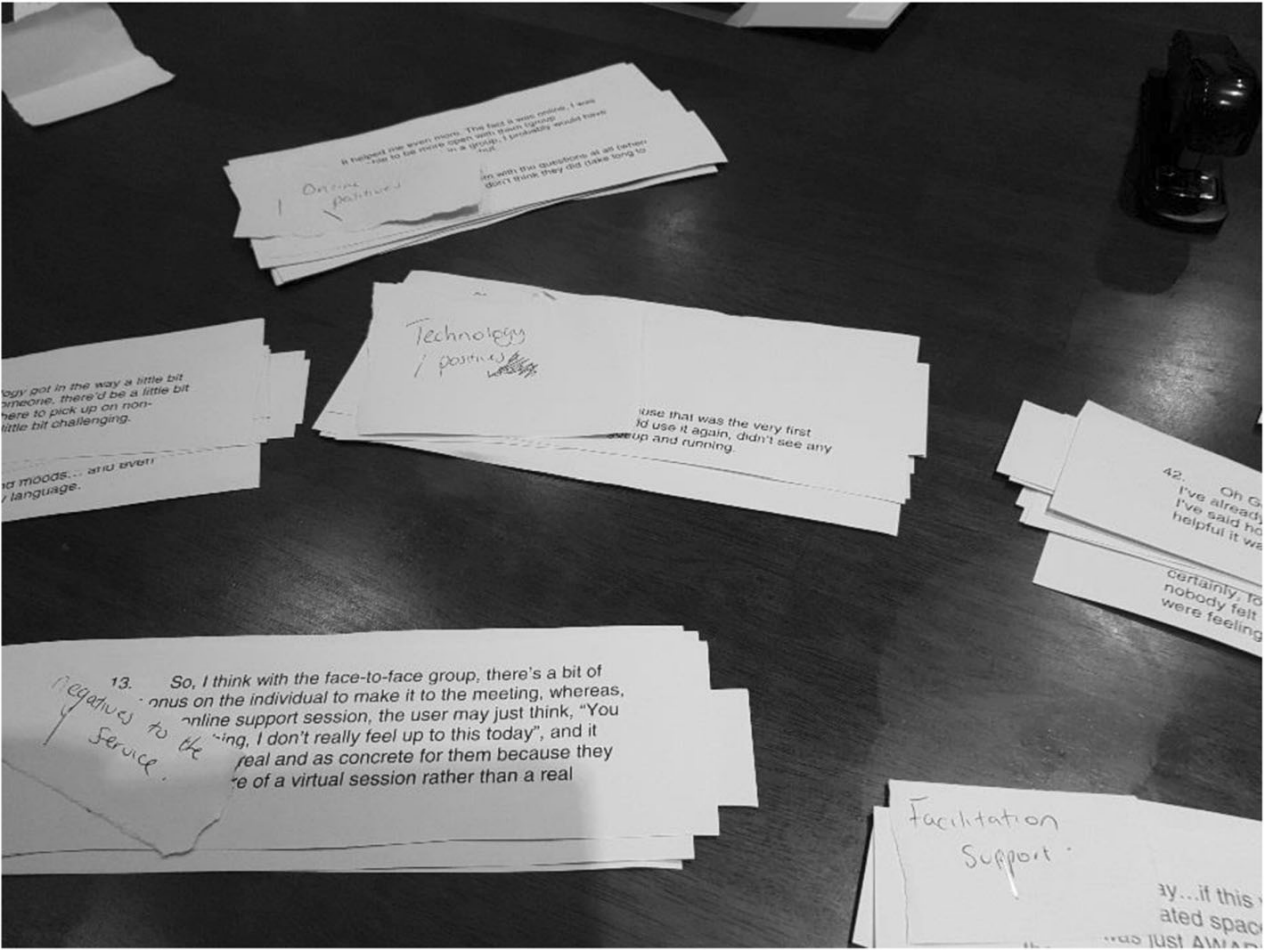
Completed piles following sorting

Data sorting was completed over a two-hour period by all nine members of the panel. Co-researchers were asked to group quotes together based on similarity with whatever criteria they felt were relevant. This task was completely independently with no input from members of the research beyond clarifying the task and queries regarding specific words or phrases. Each co-researcher produced between 3 and 12 piles of excerpts which were labelled and then stapled together (Fig. 2).

These piles were gathered by the research team and uploaded into an excel sheet wherein the following three columns were used (1) co-researcher anonymised ID; (2) quote ID; (3) pile number/label. This data was then uploaded for network analyses (step 4) which sought to create groupings based on how consistently co-researchers placed the same quotes together in a pile. Data grouping first creates a network of quotes. If any co-researcher placed a pair of quotes into the same pile, then that pair of quotes has an edge joining them in the network. The edges in the network were assigned weights, which were calculated as the inverse of the number of co-researchers who placed the pair of quotes into the same pile. For example, if two (of nine) co-researchers believed the quote ID1 and quote ID2 were sufficiently similar to be placed together, then the edge between ID1 and ID2 was assigned a weight of ½. The network was separated into groups using two well established community detection algorithms [40, 41] which attempt to maximise the edges between items in the same group and minimise the edges between groups. Network analyses was conducted using the R statistical software package [42], however the authors have developed a user friendly web-based application that will perform the network analysis on uploaded data, see http://www.ptegroups.net/main.html.

**Fig. 3:**
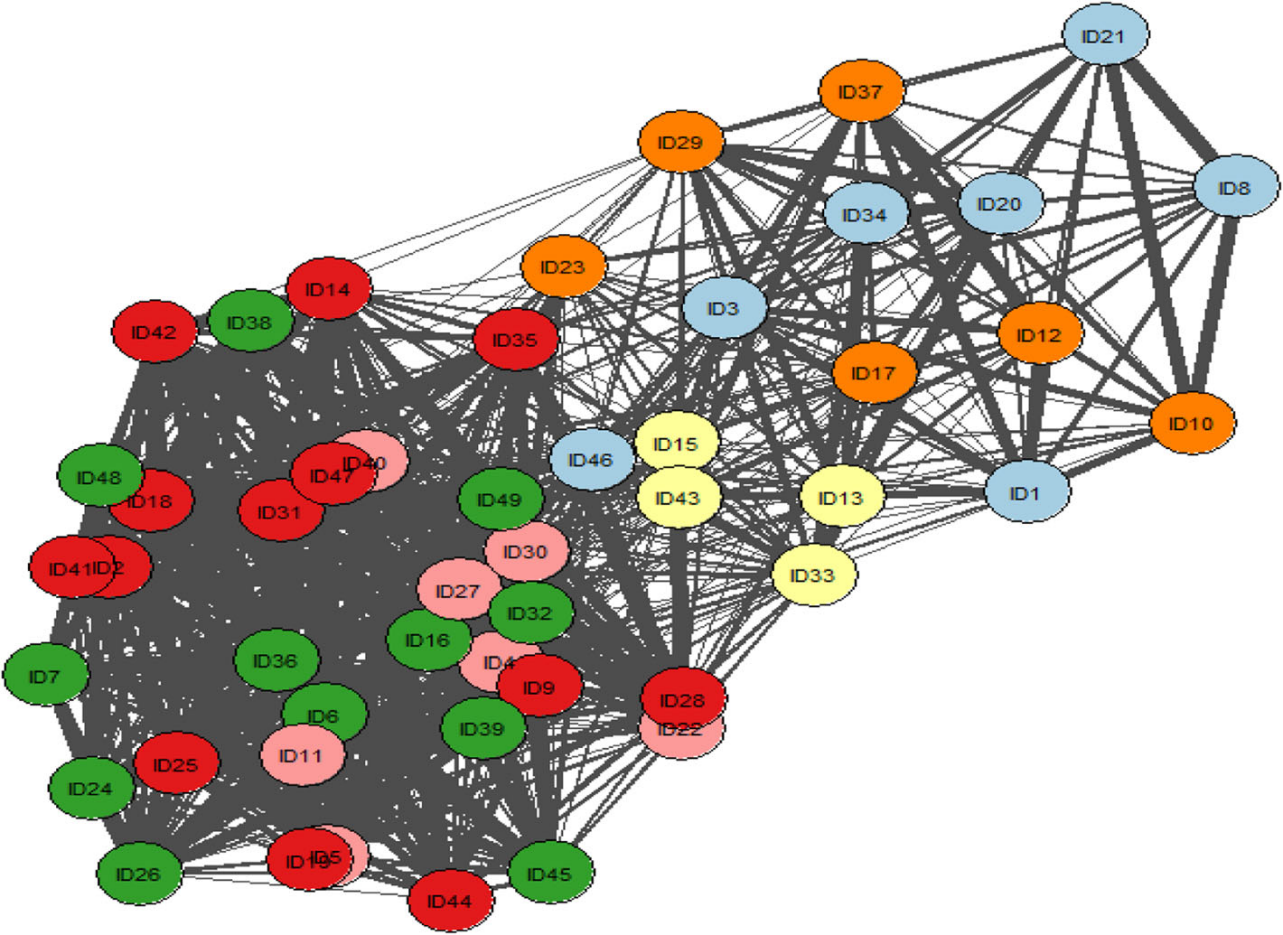
Network Diagram: The network of excerpts (shaded circles), with the thickness of the edge indicating the number of co-researchers who sorted the pair of excerpts into the same pile. The colour indicates groups identified in Step 3: Group 1 (light blue); Group 2 (green); Group 3 (pink); Group 4 (red); Group 5 (yellow) and Group 6 (orange)

Network analyses produced six core groupings. Group 1 is represented as light blue; Group 2 is green; Group 3 is pink; Group 4 is red; Group 5 is yellow and Group 6 is orange. Approximately two weeks after data sorting took place, the network diagram, along with a list of the quotes contained within each group were presented to the co-researcher panel (Step 5). They were informed that the material provided was for guidance purposes only and they could disregard any of this information at any point. Due to personal issues only six of the nine co-researchers were able to attend. Two research team members (PB and TC) also attended and the session was audio-recorded. Themes and initial codes were recorded on flipchart paper (Fig. 3).

## Results

This section presents the analysis as conducted by the co-researcher panel. It was written up by members of the research team and presented to co-researchers to ensure it accurately reflected their analysis of the data. The research team also regularly reviewed the audio recordings for accuracy. Co-researchers produced five core themes using the PTE method. These included; (1) challenges of online services; (2) benefits of accessing an online service; (3) role of technology in facilitating help-seeking; (4) therapeutic processes and outcomes within an online context and; (5) service improvements. Thematic relationships were developed within five of the six groups produced via network analysis (Step Four).

### Challenges of online services

**Fig. 4:**
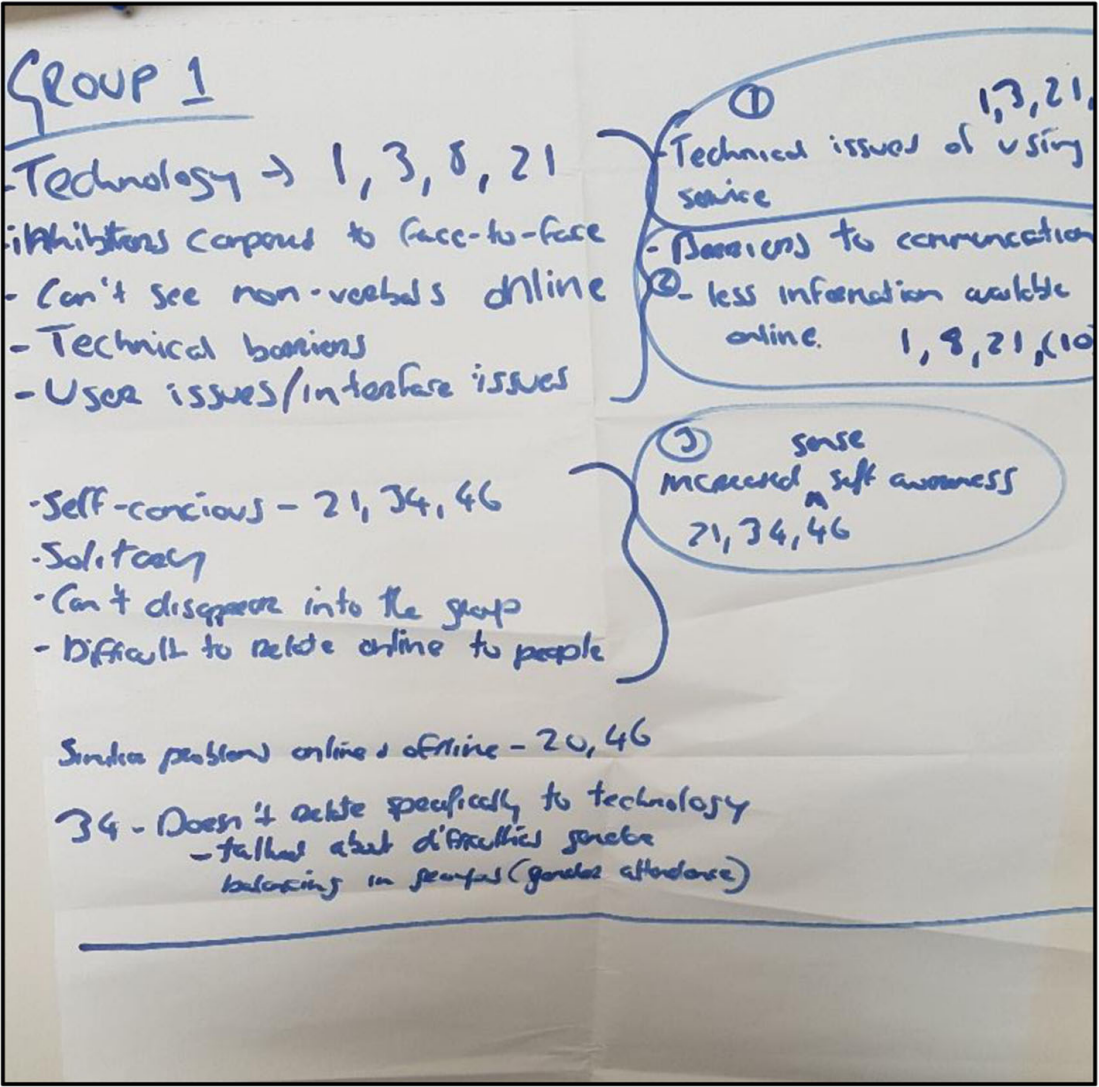
Photo of Flipchart Work from Group

This theme had three important sub-themes – (a) technical issues of physically using the service (b) barriers to communication (in-group) and; (c) increased sense of self-awareness (see Fig. 4). This first sub-theme related mostly to hardware issues, such as internet connection speed. For example, several participants noted *“the technology was just a wee bit of an issue, but … I think it probably resolved itself because I then moved into an office in our building where we have a good system” [ID03]* and *“I think, when it started, there were one or two just technical things that … weren’t a big deal but, in some way, kind of did interrupt the flow of the conversation … so, that was quite distracting” [ID01].*

The challenges didn’t stop there. Once an individual was online there were reported communication barriers. These mainly related to a lack of non-verbal communication pathways - *“so, I think it’s less easy on a videoconferencing call to actually pick up any non-verbal’s of the other people who are not talking” [ID08].* It is worth noting that both sub-themes were derived largely from the same excerpts - perhaps revealing differing views within the group or that co-researchers felt that some excerpts contained multiple meanings. In any case, this sub-theme is broadly similar to other literature in relation to online communication and ones sense of ‘presence’ [44, 45]. The final sub theme in this group was labelled as ‘increased sense of self-awareness’. One excerpt read,

*“I thought it would have been nice to maybe have a few more men that I would be able to relate to, because people like myself, obviously, we question ourselves … I think we suffer in silence, that’s the problem” [ID34].*

Originally discussed as relating to ‘group composition’, co-researchers developed (and recoded) this sub-theme by reflecting upon their own experiences. They surmised that participation in online groups might actually accentuate one’s sense of self-awareness as users had to observe each other closely in order to ‘pick up’ on important non-verbal clues. One participant stated that, *“I really found that extremely difficult … in terms of syncing the voice when we were talking … trying to be sensitive, pick up on tones and moods … and even looking at the people and their body language”* [ID21]*.* This feeling of being closely ‘watched’ may have heightened one’s sense of self within the group. While additional data is needed to support this finding, this is an interesting example of co-researchers moving from semantic to more latent level thematic analysis [46]. Rather than theorising the wider implications in relation to the literature, they reflected upon their own experiences of group therapy and thus provided a level of ‘insider’ knowledge that would have been otherwise absent.

### Benefits of accessing an online service

This theme contained two sub-themes – (a) familiarity and comfort of home environment and (b) ease of communication within online groups. Being able to join an online group from the comfort of one’s home was an important benefit for participants. Increased comfort through a familiar setting appeared to reduce anxiety e.g. *“I mainly wanted online because I don’t do very good in public, in groups … online I could do it from the comfort of my own home” [ID38].* Group facilitators also noted that,

*“There may be people who may feel more comfortable with the teleconferencing … the fact that they’re in their own home, rather than having the anxiety and … sometimes the discomfort that you can find when you have to take yourself out of that safe space and you have to go in to another space” [ID16]*

Communicating online was also felt to be easier – *“I think my part in the group was more conversational than it might be in a face-to-face group, where I probably would have said far less, largely because the face-to-face groups tend to be bigger” [ID06].* Another participant stated, *“I felt like I could open up a lot more than I would do in a room full of people. It felt a lot more comfortable for me” [ID26]*. This disinhibition effect has been noted in other studies of online behaviour [47, 48].

### Role of technology in facilitating help-seeking

This theme was developed from excerpts within group 3. Participants described how technology enabled greater flexibility when seeking help for mental health issues. This was suggested as a key reason for signing up for the service. One user noted *“it seems to work with most settings, you can just use it from your phone [ID05]*. It was also felt that the system was user friendly - *“I think it’s good that we see each other, and people who have been on the calls, not all of them have been used to Skype and yet it’s been a system that people have got used to very quickly” [ID04]*. Access to services is often viewed as a key determinant of health status [49] with analysis here suggesting that video-conferencing may support this process. Moreover, the finding appears in line with broader policy initiatives in the area [50].

Interestingly, it was felt that other excerpts contained within group 3 (ID04, ID11 and ID30) had a stronger relationship to themes developed within group 2. As such, co-researchers moved those excerpts to form a sub-theme within group 2 (ease of communication within online groups).

### Therapeutic processes and outcomes within an online context

Data revealed a strong sense that therapeutic relationships had, and could be, developed online. One participant noted, “*So, it’s been really good for me, and it’s brought me a little bit closer to other people, even though it’s online” [ID25]*. The facilitator emerged as a key factor – “*I think, (facilitator) is great … She actually comes in and she says, well, I can relate, and this is what happened to me”. I think she’s amazing” [ID28]*. The combination of group-based therapeutic approaches and relatable facilitation appeared to produce positive outcomes. For example, one participant spoke about gaining the courage to leave an abusive relationship –

*“One of the benefits was, just being able to open up to somebody, with my problems, that would listen to me and gave me some great advice... it’s actually got me out of an abusive relationship, for a start … so that’s a very, very positive thing that came out of it” [ID41]*.

It was also suggested that the online group was a potential gateway into face-to-face services as one group facilitator remarked, *“an interviewee [group user] had mentioned that …*. *she is now looking at joining the other services offered by us” [ID44].* Co-researchers grouped these together under the one theme, however they acknowledged that these could be developed further if more excerpts were available.

### Service improvements

The final core theme identified by co-researchers was ‘service improvements’. Four of the six excerpts in this group appeared particularly relevant here. For example, *“maybe a little bit of training around Skype might be useful, just to be fully familiar with it, and then just to explore, small things that might make the service a little bit better or might ensure that things are smoother” [ID12]*. Participants also felt that additional measures should be in place to support older users of the service, particularly in regard to a general awareness of technology and its various uses -

*“But I do feel though that you need to be aware of the technology, how it works - and there’s a lot of elderly men and women, they’re horrified at the prospect of having to learn something new or to maybe manage something technically” [ID37].*

In addition, they felt that ID43, which was located in Group 5, could also be used to support this theme –

*“I thought more people would have actually jumped at the chance to get into it. Unless putting a testimonial … about somebody being afraid of using Skype but then found it so beneficial or so easy to use in the end … because I think that might be what the stumbling block is” [ID43].*

## Discussion

Data analysis suggests that participants who took part in the DES project found the online groups largely beneficial. This was due, in part, to ease of access, quality of facilitation and a reduced sense of anxiety from being able to connect with others from a familiar environment (often one’s home). Qualitative data also revealed that it was easier to ‘open up’ online and therefore promoted further disclosure of problems. Advances in mobile technology also meant that accessing online groups was affordable as webcam and computer purchases were not necessary. There was a clear sense of the potential for a therapeutic alliance to develop online. This was evidenced through positive comments regarding the group facilitator as well as participants revealing improvements in their mental state. There was also evidence that online groups had given some participants the confidence to think about attending face-to-face groups whereas others had made significant life changes (leaving an abusive relationship).

Interestingly, co-researcher PTE analysis of interview data suggested that while a therapeutic process was evident, the ability of one to fully integrate into a group was unclear. Drawing upon their own experiences they felt that qualitative data revealed how a participant’s sense of self-awareness may have increased within an online group setting. This may be due to a lack of physical proximity to others or the reduced non-verbal information available. Co-researchers discussed whether an online group increased one’s sense that they were under the ‘spotlight more’ and therefore, in the case of ID34, participants would seek out others with similar profiles (e.g. males) in order to feel more comfortable. While previous evidence in this area is sparse, one interesting study by Greene and colleagues [51] did note subtle differences in group processes when using VC technology as compared to face-to-face. Nonetheless, the extent to which the data presented here supports this view is limited due to the number of quotes available. It was also acknowledged that the network diagram placed the three quotes used to generate this sub-theme quite far apart (ID21, ID34, ID46). Nonetheless, this is an interesting finding which may inform future analysis of the entire data-set.

There were a number of challenges to delivering online support groups mentioned by participants. Disruptions to internet connections at times interrupted the flow of conversations and some early difficulties getting logged on were reported. Nonetheless, interview data suggests these were relatively minor. The data suggested that improvements to the service should include some training in video-conferencing technology prior to beginning the group as well as online testimonials for previous or current users to promote engagement/recruitment. It was also considered that by only interviewing those who had completed all 8-weeks of the intervention the views of those who dropped out would have been an important addition. Overall, these findings support previous literature in the field as well as offer some interesting insights and areas for future exploration. This suggests that PTE does support user developed theme generation using qualitative data. Key learning from the qualitative data presented here is summarised as –

- The group based intervention was a convenient and valued option for participants
- Using VC technology meant that the service was accessible and relatively low cost (particularly given the developments in mobile technology)
- Online communication through a VC platform may facilitate increased disclosure of problems
- Therapeutic alliances and processes are possible using VC technology with some participants clearly stating how the service improved their mental health
- Additional training and support should be offered (pre-intervention) to improve participants early experiences of using the technology
- Additional exploration is needed regarding the level of self-awareness one feels in a group based VC environment and whether this facilitates disclosure (through disinhibition) or increases anxiety.

### Researcher reflections on co-researcher analysis

The themes and sub-themes produced by co-researchers are largely reflective of the wider literature in the field [52]. This is encouraging given that the panel were inexperienced and unfamiliar with the evidence regarding digital mental health services. There were also some new insights regarding possible online group processes that require further attention, particularly in relation to performance and the level of self-awareness one might ‘feel’ within online groups. Social presence theory [53] has been used to conceptualise how technology acts as a barrier to feeling fully ‘present’ within online communication [54]. For example, if social interaction is based on a number of verbal and non-verbal cues then by reducing the communication ‘channels’ through which these can be ‘picked up’ (i.e. radio, phone, television, skype etc.) the less ‘present’ one may feel. Lower presence may reduce the level of ‘intimacy’ felt online and impede the development of therapeutic processes. Alternatively, it could lead to disinhibition [47]. Co-researcher analysis appears to suggest the participants in the intervention were very much present when online, this may have occurred to extent to which they felt additionally exposed when it was their turn to speak. This was not discussed during the review of the qualitative data by the research team and is therefore a further avenue for exploration. Moreover, it may shed new light on other aspects of the DES study, namely issues with recruitment and retention.

As an approach to user-led qualitative analysis the authors found the time commitments fairly low and are confident all PTE Steps could be completed within as little as two days. The importance of capacity building and data sorting prior to analysis and interpretation cannot be overstated. Little moderation of group discussion was needed during Step 5 with discussion taking place organically and without prompting given the familiarity co-researchers had established with the data. The network diagram and network groupings appeared to be useful starting points and there was no evidence of one or two co-researchers monopolising the discussion or significant disagreement among co-researchers.

### Strengths and limitations

Involving those with lived experience of depression is a clear strength of this study. However, involvement alone is tokenistic and it is important that co-researchers are given the opportunity to develop skills and expand their knowledge in order to increase the quality of analysis. PTE provided the structure in which to do this and the identified themes appear both logical and insightful. Important limitations to note are that the number of quotes were from a sub-sample of qualitative data and this may have unintentionally narrowed and influenced theme generation. Moreover, the data presented was collected from those who had completed all 8-weeks of the intervention and may have been more likely to give positive feedback. There were also three members of the co-researcher panel who could not attend the data analysis and interpretation session. While the strength of this approach is the independence of the network analysis results from researcher input, the results may be influenced by these limitations and one could also consider running the process in tandem with more formal academic researcher analysis to compare findings.

The network analysis used to produce diagrams and groupings may require a certain level of specialist knowledge which may be off putting for some. In order to address this, the authors have developed a freely available, web based application [43}, that researchers can use to upload data from the sorting task (e.g. quotes ID, pile labels etc.) using an excel spreadsheet. Network analysis outputs can therefore be produced with no specialist knowledge.

## Conclusion

In conclusion, participants were largely positive regarding their experiences of using the online VC group. The ability to ‘log in’ from the comfort of one’s home combined with ease of access and moderation by experienced group facilitators appeared to be key factors. As the intervention progressed, participants found it easier to share their thoughts and feelings with others and this appeared to facilitate disclosure and ultimately aid the recovery process. The involvement of end users in data analysis provided an insider perspective and revealed interesting insights which could be followed up in subsequent research – particularly in relation to online presence and intervention recruitment and retention. However, given the limited number of quotes available, the conclusions drawn must be treated with caution and future work using the PTE method may wish to increase the number of quotes available to co-researchers while at the same time acknowledging practical issues, such as fatigue, recall and concentration.

## Data Availability

Not applicable.

## Abbreviations

DES: Developing E-health Services
PTE: Participatory Theme Elicitation
UK: United Kingdom
VC: Video Conferencing
WHO: World Health Organisation
